# Current Pharmacologic Options and Emerging Therapeutic Approaches for the Management of Ulcerative Colitis: A Narrative Review

**DOI:** 10.51894/001c.123397

**Published:** 2024-09-09

**Authors:** Sneha Annie Sebastian, Oroshay Kaiwan, Edzel L. Co, Meghana Mehendale, Babu P. Mohan

**Affiliations:** 1 Department of Internal Medicine Edgemont Medical Centre, Calgary, Canada; 2 Department of Medicine Northeast Ohio Medical University, USA; 3 Department of Internal Medicine University of Santo Tomas, Manila; 4 Smolensk State Medical University, Russia Department of Internal Medicine; 5 Department of Gastroenterology University of Utah School of Medicine, Utah, USA

**Keywords:** Ulcerative colitis, 5- Aminosalicylic acid, sulfasalazine, corticosteroids, biologics, immunomodulators, novel formulations

## Abstract

**Introduction:**

Ulcerative colitis (UC) is a chronic inflammatory bowel disorder (IBD) with periods of relapse and remission. Current advancements in clinical research have led to the development of more refined and effective medical therapy for UC.

**Summary of the Evidence:**

Traditional therapeutic agents such as 5-aminosalicylates (5-ASAs), sulfasalazine (SASP), corticosteroids, and immunomodulatory drugs have remained the gold standard for decades. However, their novel formulations and dosage regimens have changed their sequences in the medical management of UC. Several other novel drugs are in the final phases of clinical development or have recently received regulatory approval designed to target specific mechanisms involved in the inflammatory cascade for UC.

**Conclusions:**

This narrative review sought to provide a comprehensive knowledge of the potential benefits of standard and emerging therapies, including novel formulations, new chemical entities, and novel therapeutic approaches in managing UC. Keywords: Ulcerative colitis, 5- Aminosalicylic acid, sulfasalazine, corticosteroids, biologics, immunomodulators, novel formulations

## INTRODUCTION

Ulcerative colitis (UC) is a type of chronic inflammatory bowel disorder (IBD) characterized by a myriad of symptoms of varying severity that typically include abdominal pain, inability to gain or maintain weight, diarrhea, hematochezia, and fever.^1^ It is an idiopathic condition with relapses and remissions and has a higher risk of association with colonic malignancy compared to healthy individuals.^2,3^ Traditional therapy for the drug management of UC has involved the use of 5-aminosalicylic acid (5-ASA) compounds, Sulfasalazine (SASP), and corticosteroids. SASP and 5-ASAs are anti-inflammatory medications that can help induce remission and maintain clinical response in mild to moderate cases of UC.^4^ SASP has been used in the treatment of UC since the 1940s. Its chemical structure is composed of sulfonamide (sulfapyridine), and mesalazine (a 5-ASA compound) joined with an azo bond.^5^ In the colon, the azo bond gets split by bacterial action, and the sulfapyridine component gets absorbed systemically by the body, which can cause adverse effects such as nausea, fever, and headache.^1,5,6^ Mesalamine, also known as mesalazine, is a 5-ASA drug with salicylic acid substituted by an amino group at the 5^th^ position. Based on studies, 5-ASA agents are developed to overcome the adverse effects of SASP with better tolerance and fewer side effects than SASP.^5^ Evidence suggests that the newer diazo-bonded 5-ASA formulations like olsalazine and balsalazide also have better tolerability than SASP.^5^

Now, a wide range of therapies has emerged, including alpha-4-integrin blockade agents, anti-CD3 antibodies, probiotic bacterial therapy, epidermal growth factors, etc., which offer the prospect of improved dosing and compliance with traditional molecules. Also, the development of new molecules, novel formulations, and therapeutic approaches, which may deliver disease control in patients unresponsive to, or intolerant of, standard treatments, have broadened the therapeutic options of UC. These drugs have the potential therapeutic effect in treating active inflammation and preventing inflammation and neoplastic transformation.

## SUMMARY OF THE EVIDENCE

### THERAPEUTIC GOALS IN ULCERATIVE COLITIS

**Figure 1.**
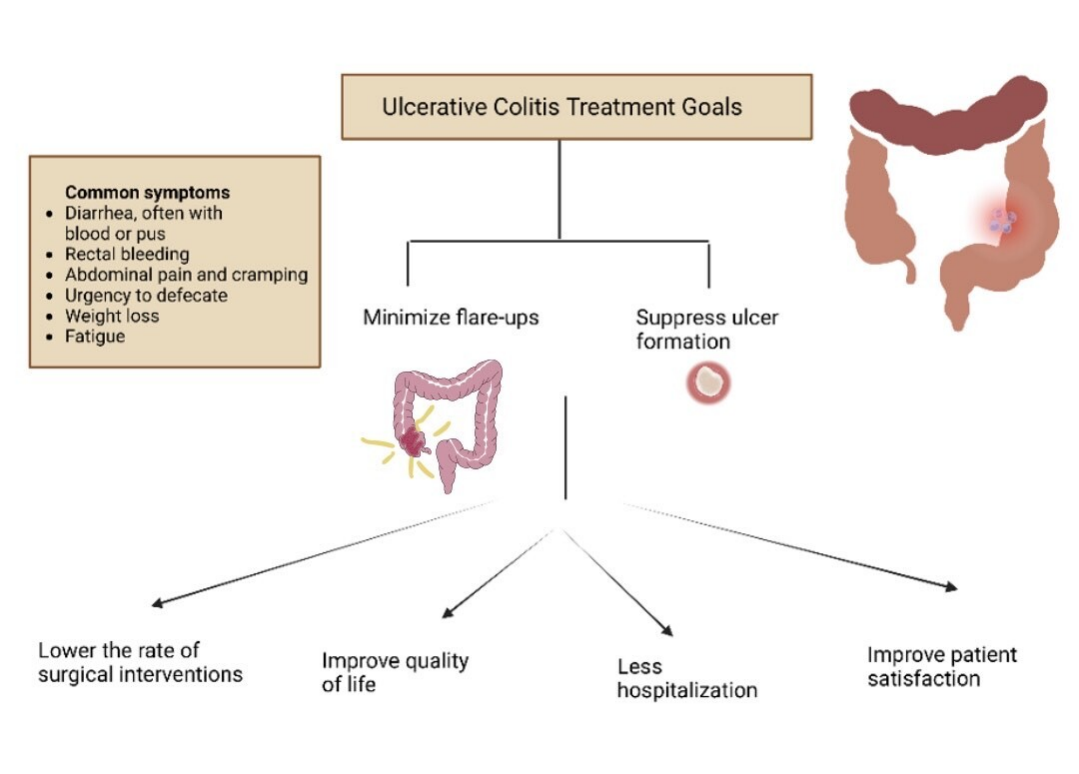
Representation of treatment goals of ulcerative colitis

The management of UC concentrates on minimizing the resurgence or exacerbation of symptoms related to the condition, limiting the necessity of using steroids, as well as safeguarding the bowel mucosa by suppressing the formation of ulcers, which would facilitate healing, thereby lowering the risk of infections, hospitalizations, indirectly promote patient satisfaction by enhancing the quality of life, and diminish the need for surgical interventions **(Figure 1)**.^7^

The remission of UC has been found to have a positive correlation with mucosal healing confirmed with endoscopic visualization, hence endoscopic scores are preferred by clinicians all around the globe to determine disease severity.^8^ Assessment of the extent and activity of UC using these scores would help in deciding the treatment options. The most often used assessments and resources for determining the magnitude of UC are a subgroup of endoscopic scores in the MAYO score and the Ulcerative Colitis Endoscopic Index of severity (UCEIS) score.^8^ MAYO ratings are preferable in the clinical context because they are simple to configure.^8,9^ However, some data suggest that UCEIS outperforms the MAYO endoscopic assessment in terms of quality and consistency.^8^ However, none of these sources address patient satisfaction, quality of life, the quantifiable plasma concentration of inflammatory biomarkers, or disease progression, including morphological disruption, recurrence, and systemic sequelae of UC.^9^ As a result, there is an increasing need to develop an assessment tool that incorporates an integrative framework for determining disease severity.

Besides Endoscopy, histopathological scoring of biopsy specimens is an additional way to derive conclusions about the severity of the disease.^8^ The presence of neutrophils in all aspects of the bowel mucosa, immune cells in the lamina propria of the colonic mucosa, damage to the mucosal cells or snaps in the mucosal surface, the appearance of granulation tissue or exudation, interruptions of colonic crypts, and a decline in the number of mucosal goblet cells are clear indicators of active disease.^10^ A remission of this condition is defined by the absence of these typical inflammatory characteristics of the existence of mucosa that is near to normal.^10^ Based on inter-rater reliability, the most generally used indices, namely the Geboes score, Nancy score, modified Riley score, and Robert’s histopathology (RHI), indicated comparable outcomes in the evaluation of disease severity.^11^ Moreover, previously, the Nancy and Geboes scores were found to be correlated to the Mayo endoscopic score and fecal calprotectin (FC) concentrations.^8^ Furthermore, investigations have revealed the existence of the histologically active disease in patients who are clinically and endoscopically in remission.^12^ Based on this premise, newer approaches are warranted to develop an inclusive tool for better judgment of the clinical states of patients with UC. Moreover, ceasing the treatment regimen in such patients may result in recurrence or flare-ups as shown by previous research.^13,14^ **Table 1.** details the summary of various assessment tools and their variables for severity scoring in UC.

**Table 1.** Summary of various assessment tools and their variables used for severity scoring in ulcerative colitis

**Assessment Tools for Determining UC Severity**	**Variables in Scoring System**
Mayo Score	Stool Frequency, Rectal bleeding, Findings of flexible sigmoidoscopy, Physician’s global assessment^15,16^
Ulcerative Colitis Endoscopic Index of Severity (UCEIS)	Vascular pattern, Bleeding, Erosions, and ulcers^16,17^
Ulcerative Colitis Overall Disease Severity Index	Mucosal lesions, Daily activity impact, CRP level, Biologic use, Recent hospitalization, Steroid use, Anemia, Frequency of stools, Albumin level, Disease extent, Nocturnal bowel movements, Anorectal symptoms, Rectal bleeding^16,18^
Montreal Classification of Extent of Ulcerative Colitis	Disease extent^16,19^
Proposed American College of Gastroenterology Ulcerative Colitis Activity Index (2019)	Stools/day, Blood in stools, Urgency, Hemoglobin, ESR, CRP (mg/L), FC (μg/g), Endoscopy (Mayo subscore), UCEIS^16,20^
Integer Score	Mean stool frequency, Colonic dilatation, Hypoalbuminemia (<30 g/l)^16^

### SULFASALAZINE IN THE INDUCTION AND MAINTENANCE OF REMISSION

SASP is a disease-modifying antirheumatic drug (DMARD) that is used to treat several inflammatory conditions like UC. The drug is metabolized into sulfapyridine and 5-ASA (mesalamine) by intestinal bacteria and further excreted by kidneys and bile. The efficiency of SASP has been studied extensively, and a comparison of the drug against other pharmacological treatment options for UC is necessary to provide the most up-to-date treatment guidelines.^21^ Yoshino et al. examined the effectiveness of SASP in treating refractory UC and demonstrated that 69.4% of patients achieved clinical remission after treatment initiation with SASP.^21^ They additionally noted that 64.3% of patients previously treated with mesalazine enemas and with sulfasalazine achieved the same remission and allowed for the discontinuation of the previously prescribed enemas.^21^ Further analysis of the relative effectiveness of the drug demonstrated improvement of symptoms during and after treatment in comparison to controls but decreased effectiveness at reducing symptoms compared with newer medications such as Mesalamine.^22^ However, additional studies reported improved efficiency and reduction in the need for steroids in patients who switched from mesalamine to SASP.^23^ These findings were further complicated by the timing and formulation of the drugs, suggesting more research is needed to make a better comparison.^23^ Furthermore, studies have demonstrated that the active ingredient of SASP (mesalamine) showed no better clinical improvement of stool qualities when compared with SASP itself, suggesting that effectiveness is comparable between the two drugs.^24,25^

### 5-AMINO SALICYLC ACID (5- ASA) MEDICATIONS IN MILD TO MODERATE ULCERATIVE COLITIS

#### Mesalamine

Newer salicylate-based drugs with fewer side effects have been developed and started using it for UC treatment lately. These are free of the sulfur component and are composed of 5-ASA without the sulfapyridine carrier molecule. Mesalamine, also known as mesalazine or (5-ASA), is one of the currently available 5-ASA-based agents used for the treatment of UC.^26^ Mesalamine acts on colonic mucosa and reduces inflammation through a diverse process which includes inhibiting the mediators of lipoxygenase and cyclooxygenase, interleukin-1, interleukin-2, and tumor necrosis factor-alpha. Also, it activates the Peroxisome proliferator-activated receptor (PPAR)-gamma, which helps in the transcription of a few key target genes such as nuclear factor B, signal transducers, and activators of transcription that help in the control of intestinal inflammation.^27^

The efficacy of mesalamine is identified as dose-dependent. Based on Kornbluth et al. and Hanauer et al. ’s study, the efficacy of mesalamine in mild to moderate disease was reported as 40-70%.^28,29^ Clinical trials by Zakko et al. in 2016 using a pooled analysis of 2 identical phase 3 randomized double-blind trials with once-daily mesalamine granules (MG) 1.5 g or placebo for up to 6 months reported that 79.4% of patients were relapse-free at six months from a pool of 373 participants versus than placebo (62.4%; P < 0.001).^30^ This study established the efficacy and tolerability of once-daily MG in preventing UC flares.^30^ In the study of Chibbar et al., significant improvement in endoscopic remission rates with reduced relapse risk has been documented in the administration of mesalamine doses of more than 2.4 g/day.^31^ 5-ASA Multi Matrix System (MMX) mesalamine, a novel, high strength (1.2 g) oral formulation was designed for once-daily dosing.^32^ It releases the active moiety throughout the colon with a favorable safety profile. Studies have reported that 5-ASA-MMX is as effective as 5-ASA enema in the treatment of mild-to-moderate, left-sided UC and considered to be the best agent for maintaining patient compliance and treatment effectiveness.^33^ In addition, once-daily administration of MMX-5ASA may lead to better adherence to therapy among patients and thus better treatment outcomes.^34^ Nevertheless, SASP is still considered the drug of choice for UC, with 5-ASAs being reserved for cases with no response to SASP treatment.

#### Olsalazine

Olsalazine, another 5-ASA, was studied mainly in patients with UC who were intolerant to SASP, and for relapse prevention.^35^ Olsalazine was developed based on the same azo-splitting principle in the colon as SASP. Olsalazine consists of two 5-ASA molecules joined by an azo bridge. Pharmacokinetic studies have shown that on oral administration there is little systemic absorption of olsalazine, and almost the whole dose passes into the colon, where the olsalazine is completely split into 5-ASA and subsequently excreted in feces and urine.^36^ Several studies have demonstrated the effectiveness of olsalazine in active mild to moderate UC, and it is also effective in the maintenance of remission of UC. Findings from both long and short-term comparative studies demonstrated that olsalazine 1 to 3g daily in divided doses improved clinical signs and symptoms of colitis in approximately 60 to 80% of patients with acute UC of mild to moderate severity, and a dosage of 1 g daily in divided doses was beneficial for the maintenance of remission of UC.^35^

The most common adverse effects of olsalazine are dose-dependent watery diarrhea and GI upset.^36,37^ This diarrhea is distinguishable from that associated with IBD by the high-water content and the absence of blood. Olsalazine-induced diarrhea usually occurred soon after initiation of olsalazine therapy or dosage increase, was more frequent with higher doses, and was usually transient.^38^ Also, dosage reduction and concomitant administration with food reduced the severity in many patients with persistent olsalazine-induced diarrhea.^37^ A study done by Ewe K et al. compared the side effects and therapeutic efficacy of SASP and olsalazine with a crossover design using SASP, 3 g/day, and olsalazine, 1.5 g/day in a total of 41 patients with mild or moderately severe left-sided colitis or proctitis documented that olsalazine is a safe and effective drug for the treatment of mild or moderately severe UC, and is comparable to SASP, though with fewer side-effects.^38^ Kruis et al. did a randomized controlled trial comparing the efficacy and tolerability of olsalazine sodium (3 g/day) with another 5-ASA formulation of enteric-coated mesalazine (3 g/day) in inducing endoscopic remission in patients with mild to moderately active UC and reported that both did not differ in inducing remission in this study group for 12 weeks of treatment.^39^

#### Balsalazide

Balsalazide is also a 5-ASA drug, a well-tolerated and effective first-line therapeutic option for patients with UC, both for active mild-to-moderate disease and as maintenance therapy to prevent disease relapse.^40^ The aminosalicylate balsalazide is a prodrug that is metabolized by bacterial azo reductases in the colon to release its therapeutically active moiety mesalazine [mesalamine or 5-ASA] and an inert carrier molecule. There is limited systemic absorption of balsalazide and its constituents, and this has a negligible therapeutic effect on drug efficacy.^40^ Clinical trials have shown that balsalazide presents with better patient tolerability in both acute and maintenance treatments of UC compared to the SASP standard formulation.^40^ A higher frequency of balsalazide can result in more rapidly symptomatic remissions than mesalamine, as proven by some studies.^41^

In regards to dosage, balsalazide 6.75 g/day was more effective than mesalamine 2.4 g/day and as effective as SASP 3 g/day for inducing remission in patients with acute UC.^42^ Moreover, complete symptom relief occurred more promptly with balsalazide 6.75 g/day than with mesalamine 2.4g/day.^42^ In long-term studies, balsalazide 2 g/day was as effective as sulfasalazine 2 g/day and balsalazide 6 g/day was as effective as mesalazine 1.5 g/day, in maintaining remission in patients with UC.^42^ The tolerability profile of balsalazide is significantly better than that of SASP; 70% of SASP-intolerant patients were able to tolerate balsalazide.^42^ The common side effects of balsalazide include headache, diarrhea, nausea, and vomiting. There were reported cases of bloody diarrhea. Overall, adverse events on balsalazide were less frequent than those on SASP.^43^ **Table 2.** provides a summary of newer 5-ASA medications, their efficacy, tolerability, and adverse effects. **Table 3.** summarizes currently available FDA-approved SASP and 5-ASA drug formulations and their indications in UC.

**Table 2.** Summary of newer 5-ASA medications, their efficacy, tolerability, and adverse effects.

**5-ASA Medication Types**	**Efficacy and Tolerability**	**Adverse Effects**
Mesalamine	Efficacy is dose-dependent. Tolerability is the same as SASP.^28,29^	Common side effects include abdominal pain, nausea, headache, and fatigue. Pancreatitis is a rare side effect.
Olsalazine	Effective drug for the treatment of mild and moderately severe UC, and is comparable to SASP.^35,37^	Common side effects are dose-dependent watery diarrhea and gastrointestinal upset. Diarrhea is non-bloody.^36,37^ It can be reduced by taking medication with food. Rare side effects are hair loss, pancreatitis, or inflammation of the tissue surrounding the heart (pericarditis).^44^
Balsalazide	Effective for active mild-to-moderate disease and as maintenance therapy to prevent disease relapse. The tolerability profile of balsalazide is significantly better than that of SASP.^39^	Common side effects of balsalazide include headache, diarrhea, nausea, and vomiting.^44^

**Table 3.** Overview of FDA-approved SASP and 5-ASA drug formulations used for the treatment of UC^45–49^

**Drug**	**Indications**
**Oral Prodrug Forms**	
Sulfasalazine (SASP)	Mild to moderately active UC Adjunctive therapy in severe UC Maintenance of remission of UC Enteric-coated tablets are indicated in patients with UC who cannot take uncoated SASP tablets because of GI intolerance
Olsalazine	Maintenance of remission of UC in patients intolerant to SASP
Balsalazide	Mild to moderately active UC in patients ≥ 5 years
**Oral Delayed-Release Forms**	
Mesalamine delayed-release tablets	Moderately active UC
Mesalamine delayed-release capsules	Mild to moderately active UC in patients ≥ 5 years Maintenance of remission of UC in adults
Mesalamine Multi Matrix System (MMX) delayed-release tablets	Mild to moderately active UC Maintenance of remission of UC
Mesalamine extended-release capsules	Mild to moderately active UC Maintenance of remission of UC in adults
**Rectal Forms**	
Mesalamine enemas	Mild to moderately active distal UC, proctosigmoiditis, or proctitis
Mesalamine enemas sulfite-free	Mild to moderately active distal UC, proctosigmoiditis, or proctitis
Mesalamine suppositories	Active ulcerative proctitis

### ROLE OF CORTICOSTEROIDS

Corticosteroids in the treatment of UC mainly dwell in the induction of remission, after which the drug can be tapered and discontinued (over 8-12 weeks) due to its extensive side effects. In a meta-analysis by Ford et al., standard glucocorticoids were superior in achieving UC remission compared to placebo.^50^ Some formulations of glucocorticoid used in UC include hydrocortisone suppository, hydrocortisone aerosol foam 10%, hydrocortisone enema, prednisone, budesonide, methylprednisolone, etc. In mild to moderate UC, patients who cannot tolerate initial therapy, like 5-ASA agents, are treated with glucocorticoid. The formulation of glucocorticoid depends on the disease severity, patient risk (low versus high), disease extent, and therapy intended to achieve, that is, induction of remission. For example, in ulcerative proctitis (involvement of ≤18cm from the anal verge), a glucocorticoid suppository can be used. For ulcerative proctosigmoiditis (>18 cm from the anal verge), glucocorticoid foam or enema can be used. For left-sided or extensive colitis, topical glucocorticoid therapy (suppository, foam, enema) is combined with 5-ASA agents.^50^ If this combination is unsuccessful, budesonide can be added with escalation to prednisone (systemic glucocorticoid therapy) or a biological agent if necessary. In moderate to severe UC, the first-line therapy for induction is a glucocorticoid or biological agent. In acute severe UC, the initial therapy in hospitalized patients is usually intravenous glucocorticoid with the expectation of clinical improvement in three to five days. Intravenous steroids have been shown to induce remission in up to 70% of patient cases.^51^

Oftentimes, UC is defined based on the response of the disease to glucocorticoid therapy. In glucocorticoid-responsive disease, UC is termed “responsive” if there is a clinical response to oral prednisone within 30 days. In glucocorticoid-dependent disease, UC is termed “dependent” if glucocorticoid cannot be tapered to less than 10mg daily within three months of initiating the therapy or occurrence of relapse within three months of stopping the glucocorticoid therapy. In the case of glucocorticoid-refractory disease, UC is termed “refractory” if there is no clinical improvement in response to oral prednisone.^51^

Generally, prednisone begins to show improvement in symptoms within a week and can be tapered by increments of 5mg or 10mg. However, the response to corticosteroid therapy is extremely individualized, inducing corticosteroid dependence or refractories at which point, other modalities are employed. Therefore, more studies need to be done to explore the therapeutic response to corticosteroids, especially concerning race or genetic factors, which can improve the course of treatment for UC. Due to its inhibitory effects on the immune system, corticosteroids increase the infection risk. Short-term side effects of corticosteroids include hyperglycemia, hypertension, hematological changes, pancreatitis, cutaneous effects, and neuropsychological effects. Long-term side effects of corticosteroids include osteoporosis, adrenal insufficiency, growth suppression, hyperlipidemia, aseptic joint necrosis, congenital malformation, etc. Therefore, long-term use of more than eight weeks of corticosteroid is avoided due to extensive glucocorticoid side effects.^50,51^

### IMMUNOMODULATORS

As the name suggests, immunomodulators act by modifying the immune system activities leading to a decreased inflammatory response. In the treatment of UC, immunomodulators are sometimes referred to as “steroid-sparing drugs” as they decrease the long-term need for steroids. They are often used in combination with biological agents to prevent antibody formation, thereby, further augmenting the action of biologics. They are used mainly for the treatment of moderate to severe UC or mild to moderate UC that is unresponsive to 5-ASA. They are less likely to induce remission and are primarily used as maintenance therapy. These include azathioprine, 6-mercaptopurine, methotrexate, and cyclosporine.^20^

Azathioprine (AZA) and 6-mercaptopurine (6-MP) are thiopurines with a slow onset of action, and the therapeutic response is seen around three to six months. Therefore, they are given in conjunction with corticosteroids or biologics. ^20^ Before starting thiopurines, patients should be assessed for their thiopurine methyltransferase enzymatic activity to know how well the drug will be metabolized in the patient’s body and to modify the initial dose accordingly. Thiopurines should be avoided in patients who lack the activity of this enzyme. Furthermore, thiopurines are associated with lymphoproliferative diseases (e.g. hepatosplenic T-cell lymphoma, Non-Hodgkin’s lymphoma) and nonmelanoma skin cancer. According to the American Gastroenterological Association (AGA), thiopurine is mainly recommended for moderate to severely active UC compared to placebo or corticosteroid use.

Methotrexate is mainly used for the maintenance of remission when used in combination therapy with biologic agents (like infliximab). However, after the development of more advanced biologic agents, it is less likely to be used for the treatment of UC. The Methotrexate Response in Treatment of Ulcerative Colitis (MERIT-UC) trial showed that compared to placebo, parenteral methotrexate showed no benefit in maintaining remission after steroid induction. ^20^ Therefore, according to AGA guidelines, methotrexate is nowadays not recommended for maintenance in patients with moderate to severely active UC in remission. Cyclosporine A is a calcineurin inhibitor that blocks T-cell activation and interleukin-2 (IL-2) transcription by binding to the cyclophilin. It is not routinely used in the treatment of UC because of its severe toxic effects, especially nephrotoxicity. Furthermore, it was used as a second-line agent in hospitalized patients with severe acute UC who failed intravenous corticosteroid treatment. However, infliximab is increasingly replacing cyclosporine as the preferred second-line agent in this patient population. Tacrolimus is another calcineurin inhibitor that blocks T-cell activation and IL-2 transcription by binding to the FK506 binding protein. It has a role in attaining clinical remission in severe colitis patients, but its utility in maintaining remission or preventing surgery is limited. ^20^

Immunomodulators present with severe side effects, mainly hepatotoxicity and myelosuppression. They require strict monitoring via blood counts and chemistries. With the advancement in medicine and the introduction of biologic agents, the role of immunomodulators in the treatment of UC is being questioned increasingly, especially as monotherapy. Their role as monotherapy has diminished in North America. More research is under way to assess the role of immunomodulators in UC.

### BIOLOGICS

Biologic agents are crucial for the treatment of moderate to severe UC. The choice of biologic agent depends on the patient’s preference, cost of the drug, availability of the drug, patient’s past medical history, provider preference, etc. Some biologic agents used in the treatment of UC include:

#### Tumor Necrosis Factor (TNF) Alpha Inhibitors

Infliximab, Adalimumab, and Golimumab are the common TNF alpha inhibitors used for treating UC. These drugs are monoclonal antibodies that act against the TNF-alpha either by neutralizing its biological activity or by interfering with the TNF-alpha attachment to its receptor site. These drugs are increasingly being used for moderate to severe UC. Furthermore, in hospitalized patients with acute severe UC, infliximab is an alternative initial therapy for patients who do not respond to intravenous glucocorticoid therapy within three to five days. Patients who do not respond to infliximab despite adequate trough levels can be switched to a different biologic class (eg. Ustekinumab). Some side effects associated with TNF-alpha inhibitors are increased susceptibility to infections, like mycobacterium tuberculosis, bacterial, viral, or fungal. Although TNF-alpha inhibitors present an increased risk of infection, several researchers have been looking to quantify the risk of infection, and outweigh it with the benefit produced by the therapy. Singh et al. compared corticosteroids with TNF-alpha inhibitors and concluded that the risk of serious infection was not significantly different from prolonged corticosteroid use; however, the former is associated with a lower mortality rate. Other side effects include malignancy (eg. non-melanoma skin cancer, lymphoid malignancy), demyelinating central nervous system diseases, neutropenia, worsening or new onset of heart failure, injection site infections, transfusion reactions, or cutaneous reactions. Therefore, contraindications to the use of TNF-alpha inhibitors include an active infection (bacterial, fungal, herpes zoster, HBV, HCV, etc), active or latent TB, malignancy, demyelination disease, non-healed infection skin ulcers, or history of heart failure. ^52^

#### Anti-Integrin Molecules

Vedolizumab was FDA-approved in 2014 for the treatment of moderate to severe UC. This drug acts by inhibiting the action of integrin, which is a protein involved in neutrophil function and migration by interacting with cellular adhesion molecules to allow for leukocyte adhesion to that vessel wall. Vedolizumab inhibits a special integrin, alpha-4-beta-7 integrin, that is found predominantly in the gastrointestinal tract and leads to local immunosuppression in the gastrointestinal tract. Therefore, if a patient has any recent infection, or malignancy, or elderly (>65), vedolizumab is usually the preferred drug of choice for UC due to its minimal effect on the immune system and decreased risk of infections compared to TNF-alpha inhibitors.^52^

#### Janus Kinase (JAK) Inhibitors

Tofacitibin and Upadacitinib are JAK inhibitors, which act by inhibiting the JAK kinase, thereby inhibiting the cytokine and growth factor receptor signaling. They are mainly used for moderate to severe UC unresponsive to anti-TNF alpha agents (patients failed at least one or more TNF inhibitors before). Tofacitibin is associated with an increased risk of thromboembolic events, herpes zoster infection, and increased cholesterol levels. Therefore, it is contraindicated in patients with a history of thromboembolic disease, active or serious infection, and severe liver impairment. Due to its severe side effects, FDA issued a black box warning in 2019 for tofacitinib to be limited to patients who experience severe side effects with conventional therapies.^52^

#### Anti-Interleukin (IL) Agents

Ustekinumab is a monoclonal antibody against the p40 subunit of both IL-12 and IL-23, which is needed for Th17 and Th1 differentiation involved in the pathogenesis of UC. It was FDA-approved in 2019 for the treatment of moderate to severe UC. Researchers are concluding the effectiveness and safety profile of Ustekinumab in treatment-refractory UC patients.^53^ Some of the adverse effects include an increased risk of infection like tuberculosis and malignancy.

#### Sphingosine 1-Phosphate (S1P) Receptor Modulators (S1PR)

Ozanimod is the first (S1PR) modulator approved for moderate to severe UC in patients who failed to respond to conventional therapies. By modulating the receptor, the drug reduces the lymphocyte movement from the lymph nodes to the site of the inflammation intestine. It is contraindicated in patients with a cardiovascular history (myocardial infarction, stroke, heart failure, or unstable angina) or severe untreated sleep apnea.^53^ The most commonly reported side effects include anemia, headache, and nasopharyngitis. Initial dosing for biologic agents is listed in **Table 4.**

**Table:** Table. 4 Initial dosing and administration for biologic agents for moderate to severe active UC.^52,53^

**Drug**	**Phase**	**Frequency**	**Administration**
Infliximab (Remicade) FDA-approved in 1998	Induction	Week 0,2,6: 5 mg/kg	Intravenous
	Maintenance	5 mg/kg every 8 week thereafter	
Adalimumab (Humira) FDA-approved in 2012	Induction	Week 0: 160 mg Week 2 (Day 15): 80 mg	Subcutaneous
	Maintenance	Week 4 (Day 29) and thereafter every other week: 40 mg	
Golimumab (Simponi) FDA-approved in 2013	Induction	Week 0: 200 mg Week 2: 100 mg	Subcutaneous
	Maintenance	Week 6: 100 mg if body weight ≥80 kg or 50 mg if body weight <80 kg every 4 weeks	
Vedolizumab (Entyvio) FDA-approved in 2014	Induction	Week 0,2,6: 300 mg	Intravenous
	Maintenance	300 mg every 8 weeks thereafter.	
Tofacitinib (Xeljanz) FDA-approved in 2018	Induction	IR: 10 mg twice daily for 8 weeks. ER: 22 mg once daily for at least 8 weeks	Oral
	Maintenance	IR: 5 mg twice daily ER: 11 mg once daily	
Upadacitinib (Rinvoq) FDA-approved in 2022	Induction	45 mg once daily for 8 weeks	Oral
	Maintenance	15 mg once daily	
Ustekinumab (Stelara) FDA-approved in 2019	Induction	≤55 kg: 260 mg single dose >55 - 85 kg: 390 mg single dose >85 kg: 520 mg single dose	Intravenous
	Maintenance	90 mg every 8 weeks	Subcutaneous
Ozanimod (Zeposia) FDA-approved in 2021	Induction	Day 1-4: 0.23 mg once daily Day 5-7: 0.46 mg once daily	Oral
	Maintenance	Day 8: 0.92 mg once daily	

### NOVEL THERAPY

#### Apheresis therapy

Apheresis means “taking away or purifying”. It is an extracorporeal vein-to-vein treatment to eliminate abnormal cells and administer plasma or selective cells into the blood. This eliminates the leukocytes from the body, thereby blocking the inflammatory response. The proposed treatment modalities in UC are granulocyte-monocyte-apheresis (GMA) and leukocytapheresis (LCAP). GMA is filled with cellulose acetate beads and mainly removes granulocytes and monocytes, while LCAP is filled with non-woven polyester fibres and mainly removes leukocytes and platelets. Research has shown its efficacy in mild to moderate UC and refractory UC (steroid-dependent UC or biologic/immunologic resistant UC or lost their response to biologics) for remission induction.^54^

#### Probiotics, Prebiotics, Synbiotics, and Postbiotics

Researchers began to explore the use of probiotics, prebiotics, and symbiotics in treating UC, as the presence of microbiomes has been known to regulate gut physiology and immune function. Some common probiotics with beneficial health effects are Lactobacillus strains, Enterococcus faecium, Pediococcus, Saccharomyces boulardii, etc. These strains help increase the production of immunoglobulin A (IgA), stimulate signaling proteins, and reduce the secretion of pro-inflammatory cytokines. However, some side effects of the probiotics include systemic infections, overstimulation of the immune system, gene transfer, and other gastrointestinal side effects. Studies on the use of probiotics in IBD have been going on since 1997. However, the benefit of probiotics in inducing or maintaining remission is still being researched. The randomized controlled trials that have been conducted are inconsistent, and lack validity and quality. AGA has no recommendations for the use of probiotics in patients with mild to moderate UC.^55^ Probiotics have no side effects, however, if they are used instead of conventional therapies, they are at risk of harming the patient. Recently, clinical studies are reporting the therapeutic effect of VSL#3 in mild to moderate.^56,57^ VSL#3 is a commercial probiotic mixture consisting of eight bacterial strains. It also has a synergistic effect when combined with conventional therapies like 5-ASA or Balsalazide. However, more research is still needed to determine the efficacy of probiotics in UC.

#### Fecal microbiota transplantation (FMT)

Fecal microbiota transplantation (FMT) is an emerging therapeutic approach for UC. This treatment involves transferring stool from a healthy donor to the patient’s gastrointestinal tract to help rebalance gut microbiota and potentially alleviate UC symptoms.^58^ Gut microbiota dysbiosis plays a crucial role in the development of UC. This condition is often characterized by an abnormal distribution of gut microbiota, with decreased biodiversity and a lower abundance of beneficial intestinal microorganisms. In UC patients, dysbiosis typically results in a reduced proportion of Firmicutes and an increased proportion of Proteobacteria.^59^ FMT aims to address these imbalances by transferring fecal bacteria from a healthy donor to the recipient, thereby enhancing the diversity and abundance of the gut microbiota.^59^

A recent meta-analysis of 13 randomized controlled trials (RCTs) involving 293 patients undergoing FMT for UC revealed that FMT was linked to significantly higher rates of clinical remission (RR = 1.73) and endoscopic remission (RR = 1.74) compared to the control group. Additionally, the study found no significant differences in adverse reactions between the FMT and control groups.^60^ Studies have explored the use of FMT in patients with acute severe ulcerative colitis (ASUC). A study reported that a patient with ASUC, who was undergoing treatment with infliximab, azathioprine, and mesalamine but did not respond to steroids by day 3, received FMT.^61^ The patient subsequently achieved both clinical and endoscopic remission.^61^ Regarding its safety profile, post-FMT, some individuals experience mild and transient adverse effects, such as diarrhea (10%) and abdominal discomfort, including pain, cramps, or bloating (7%).^62^ Serious adverse events, including infections or fatalities, have been observed in up to 1.4% of FMT procedures, primarily occurring in patients with existing mucosal damage, such as ulcers or erosions.^62^ Despite the potential of FMT for treating IBD, more RCTs are necessary to verify its efficacy and safety. Subsequent studies should focus on optimizing FMT procedures, identifying the most suitable donor criteria, and exploring the long-term safety and effectiveness of FMT in UC management.

## CONCLUSIONS

As a chronic illness, the main treatment goal in UC is to minimize the risk of flare-ups and suppress the formation of ulcers. UC necessitates long-term treatment to maintain a remission state. As a result, the need for careful evaluation of the medications to be rendered to patients considering the efficacy, tolerability, adverse effects, and cost-effectiveness is significant. It requires further investigations and prospective clinical trials by the scientific world for an increased understanding of pharmacogenomics, biomarkers, and clinical features that identify subpopulations of patients who will best respond to specific medications to provide care tailored to individual patients.

### Declaration of Interest

None
